# Effects of Recreational Camping on the Environmental Values of National Parks in Sri Lanka

**DOI:** 10.21315/tlsr2021.32.3.7

**Published:** 2021-09-30

**Authors:** Shashini Tara Mallikage, Priyan Perera, David Newsome, Rangika Bandara, Greg Simpson

**Affiliations:** 1 Department of Forestry and Environmental Science, University of Sri Jayewardenepura, Gangodawila, Nugegoda, Sri Lanka; 2 Environmental and Conservation Sciences, College of Science, Health, Engineering, and Education, Murdoch University, South St., Murdoch WA 6150, Australia; 3 Department Zoology and Environmental Management, University of Kelaniya, Kelaniya, Sri Lanka; 4 Centre for Sustainable Aquatic Ecosystems, Harry Butler Institute, South St., Murdoch WA 6150, Australia

**Keywords:** Sustainable Tourism, Camping, Recreation Ecology, Biophysical Impacts, Ecotourism

## Abstract

Camping is a popular activity in the contemporary nature-based tourism domain and rapidly gaining momentum as a key recreational activity in Sri Lanka’s national parks (NPs). Recreational uses such as camping in natural areas can induce significant and often localised resource impacts that can affect soil, vegetation, wildlife and water, with the severity of such impacts varying according to the intensity of use. Hence, monitoring of the biophysical conditions of campsites has become an important component in the reserve management agenda in many places, especially in developed countries. To the best of our knowledge, this is the first study to examine the biophysical impacts associated with the recreation ecology of camping in Sri Lanka. Ten campsites from three dry zone NPs were selected to assess biophysical impacts of camping activities. Field measurements were based on the fixed radial transect method. Gathered data included the total area of the campsite, erosion potential measured as the area of exposed soil (devoid of vegetation or organic litter), number of exposed roots and human damage to trees, number of fireplaces/ fire scars on the ground, visual counts of litter, soil compaction measured by penetrometer, loss of woody debris. This study reports significant levels of environmental degradation related to all the indictors of biophysical impacts at both high and low use campsites. There was no evidence for any difference in the level of environmental degradation associated with high and low use campsites. The loss of natural values associated with campsites negatively impacted visitors’ nature-based experience. These findings highlight the importance of managing biophysical impacts in campsites to provide a high-quality visitor experience, while sustainably managing tourism activities in NPs.

**Highlights**

- Significant levels of environmental degradation evident at campsites due to biophysical impacts of human use and recreation were observed irrespective of the level/frequency of use.
- The loss of natural values associated with campsites negatively impacts visitor experience.
- Park managers need to ensure strict enforcement of visitor management policies, education and awareness to minimise the negative biophysical impacts on campsites.

## INTRODUCTION

The use of natural areas for recreation is a growing global tourism phenomenon (Fredman & Tyrväinen 2010; Newsome *et al*. 2013; Margaryan & Fredman 2017; Marasinghe *et al*. 2020). This evolving trend places natural area tourism in a strategic position to positively support biodiversity conservation and sustainable management of protected areas, especially in the biodiversity-rich tropics (Perera *et al*. 2015; Marasinghe *et al*. 2020). National Parks (NPs) of Sri Lanka have become prime nature-based tourism destinations for both international and domestic tourists (Perera *et al*. 2012; Senevirathna & Perera 2013; Marasinghe *et al*. 2021). At the same time, escalating visitor numbers at popular NPs have led to concerns over potential negative impacts of tourism and recreation occurring within protected areas (Smith & Newsome 2002; Pickering 2010; Newsome *et al*. 2013; Prakash *et al*. 2018).

Recreational activities in natural areas can affect a range of environmental components including soils, vegetation and wildlife (Rahman & Vacik 2009; Pickering 2010; Alwis *et al*. 2016; Marion *et al*. 2016; Marasinghe *et al*. 2021). Although the total area allocated for recreation and tourism infrastructure may be relatively small compared to the total area of a park, the impacts at such sites can be severe and often permanent (Pickering 2010; Zhong *et al*. 2011; Newsome *et al*. 2013; Eagleston & Marion 2017).

With the rising demand for natural area tourism in Sri Lanka, the demand for alternative accommodation choices has also increased (Sumanapala *et al*. 2015, 2017; Simpson *et al*. 2020). The tourist preference to stay in temporary structures such as camping tents, and directly experience nature is becoming more popular worldwide, though these accommodation forms can vary from luxurious to basic. As such, designated campsites have become an essential component in designing protected area visitor facilities where recreation is deemed an important public use. A growing body of literature on biophysical impacts of camping activities has reported negative impacts under various use levels and according to the environmental context as to where campsites are located, with extensive research having been undertaken in the U.S. and Australian nature-based parks, compared to other regions of the world (Smith & Newsome 2002; Cole 2003; Buckley 2005; Pickering 2010; Marion 2016; Arredondo *et al*. 2021).

Camping activities can induce significant and often localised resource impacts that can affect soil (compaction and erosion, ground exposure, changes to the hydrology of site), vegetation (loss of ground vegetation and seedlings, trampling, change in species composition, spread of invasive plants), wildlife (habitat alteration, disturbance to wildlife), and water (increased turbidity, contamination with human faecal matter) with severity of such impacts varying on the use level (Smith & Newsome 2002; Cole 2004; Cole & Monz 2004; Reid & Marion 2005; Growcock 2005; Farooquee *et al*. 2008; Monz *et al*. 2010; Newsome *et al*. 2013 Marion *et al*. 2016; Lawrence 2018; Eagleston & Marion 2018). Past research further suggests a curvilinear relationship between the level of use and level of many impact variables, with the majority of impacts occurring during the period of initial use (Growcock 2005; Marion 2016). Visitor group size can also influence the area of damage and exert campsite expansion pressure with larger parties often causing a disproportionate amount of damage (Marion & Farrell 2002). Furthermore, the ongoing proliferation of both formal and informal campsites associated with increasing visitation and heavy use of natural areas in the world is believed to be a major contributing factor to the increase in the total campsite impact (D’Antonio *et al*. 2013; Marion *et al*. 2018).

While campsite impacts do not threaten the ecological integrity of the entire area, they can result in serious localised resource damage and potentially affect the quality of visitor experience (Farrell *et al*. 2001; Smith & Newsome 2002; Daniels & Marion 2006). As such, monitoring both the number and condition of campsites has become an important component in protected area management agendas. Though there are a number of indicators that can be used to identify the degradation of the campsite environment, changes to vegetation and soil parameters are frequently assessed because of their importance for long-term ecosystem function and ease of detection, in association with a longer time lag to recover to pre-disturbance conditions once altered (Cole & Monz 2004; Newsome *et al*. 2013). Such impacts can be measured using a range of parameters including soil compaction, penetration resistance, tree damage, root exposure, plant species composition and extent of the exposed area in the campsite (Lawrence 2018).

Other biophysical parameters often considered in assessing campsite impacts include the loss of woody debris and littering (Smith & Newsome 2002). As suggested by Eagles (2001), the success of nature-based tourism operations is highly likely to depend upon the level of environmental quality and suitable levels of consumer service. Long-term degradation of campsites due to biophysical impacts of visitation can negatively affect visitor experience (Marion & Farrell 2002; Manning *et al*. 2004; Deng *et al*. 2003; Daniels & Marion 2006). Hence, contemporary natural area management places much emphasis on the management of site impacts (Farrell *et al*. 2001; Newsome *et al*. 2013). Many scholars have attempted to understand the effect of biophysical impacts in wilderness recreational sites on visitor experience. Some studies suggest that although visitors notice the biophysical impacts on sites, these impacts do not necessarily detract visitors’ overall outdoor experiences (Farrell *et al*. 2001; Deng *et al*. 2003; Dorwart *et al*. 2009). Nonetheless, such studies acknowledge that visitors are sensitive and concerned about biophysical impacts of obvious damage, though visitor judgements on such impacts can be affected by their visual sensitivity and social elements such as attitude, knowledge and behaviors (Deng *et al*. 2003). Furthermore, an understanding of visitor perceptions on biophysical impacts in recreational sites can provide valuable insights for park managers to formulate appropriate management strategies to counter the problem (Smith & Newsome 2002; Deng *et al*. 2003; Smith *et al*. 2012; D’Antonio *et al*. 2013; Lawrence 2018).

In the Sri Lankan context, camping is becoming an increasingly popular activity in NPs; however, no previous studies have specifically attempted to assess the environmental impacts and social aspects of campsite use. Previous literature has suggested that the biophysical impacts on campsites tend to vary based on the degree of usage (Growcock 2005; Newsome *et al*. 2013). Only a few studies thus far have attempted to assess the biophysical impacts of camping in the Asian tropics (Rahman & Vacik 2009). As the majority of published recreation ecology research is from the U.S. and Australia, protected area managers and researchers in Tropical Asia (after Marasinghe *et al*. 2020) have frequently had to rely on findings drawn from research conducted in alpine or temperate settings. However, due to the variability in environmental conditions, the magnitude and level of use, site specific physical developments at campsites, and campsite visitor characteristics (such as visitor behaviours, attitude, and environmental orientation) such research findings may have some limited applicability in the Tropical Asian context. Furthermore, several authors have reported on the importance of demonstrating the local relevance of research and management practices in order to sustain or enhance nature-based tourism experiences (Bandara 2009; Fernando & Kaluarachchi 2016; Simpson *et al*. 2020; Marasinghe *et al*. 2021). To address this gap in the literature, this article reports the first attempt to assess and quantify the biophysical impacts of campsites in selected NP of Sri Lanka by using approaches that have been successfully applied in the North American and Australian contexts.

## MATERIALS AND METHODS

### Study Sites

**Figure 1:**
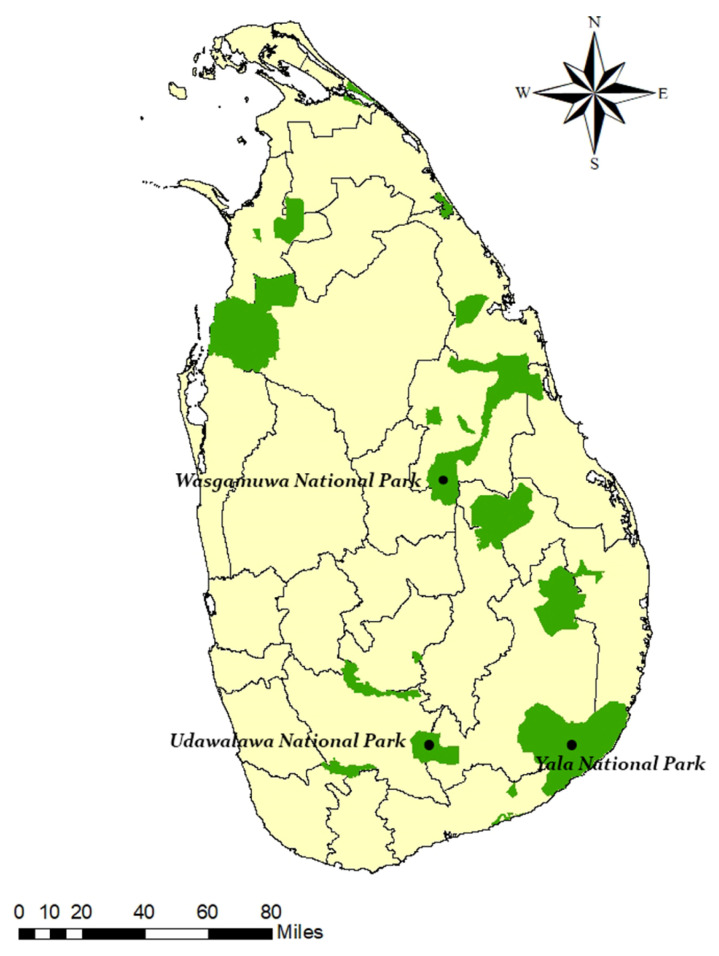
Locations of the study sites; three National Parks in the dry zone of Sri Lanka frequently visited by campers (Source: DWC 2016)

Three NPs predominantly falling within the dry climatic zone of Sri Lanka; Yala, Udawalawe and Wasgamuwa National Parks were selected as the study locations (Fig. 1). The selection of study sites was based on the annual visitation statistics (Sri Lanka Tourism Development Authority (SLTDA) 2015) and monthly booking information available at the Department of Wildlife Conservation Sri Lanka (DWC). Of the selected NPs, camping sites in Yala NP has the highest demand followed by Udawalawa and Wasgamuwa NPs (DWC 2016). A total of 10 active campsites from the three NPs were selected for the assessment of biophysical impacts. All selected campsites were located on river/stream banks with the surrounding environment comprising riverine vegetation. Each site was allocated a unique identification code for the ease of identification, interpretation, and presentation of results (Table 1).

### High Use and Low Use Campsites

Average monthly occupancy of each campsite was calculated based on the number of bookings and duration of stay. Accordingly, campsites which were occupied for at least seven days (one week or more) per month were classified as “high” use and campsites that were occupied less than seven days in a typical month were considered “low” use sites for the purpose of this study (Table 1). Park management regulations and visitor policy stipulate that only a single group of campers with a maximum of 10 individuals are allowed to occupy each campsite.

### Campsite Features and Facilities

**Figure 2:**
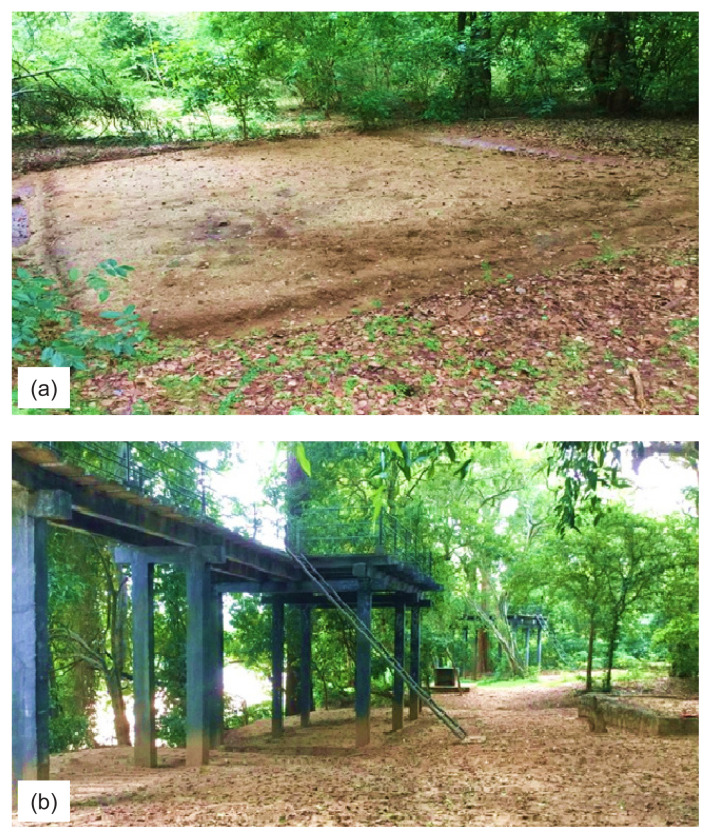
Undeveloped and developed campsites: (a) A typical undeveloped campsite with a designated elevated tenting area, Udawalawa NP; (b) A developed campsite with elevated camping platforms and other supporting structural facilities, Wasgamuwa NP.

Biophysical impacts on campsites can vary based on the degree of planning for recreational camping and provision of facilities for different activities by park management. Based on the type of development and infrastructure facilities available, campsites were classified as “developed” and “undeveloped” (Table 1). For the purposes of this study, these two categories were defined as follows. Undeveloped campsites are campsites with basic facilities, including an area devoid of vegetation purposefully cleared by park management, with a toilet provided. Some undeveloped campsites may include slightly elevated tent pads constructed with earth [Fig. 2(a)]. Developed campsites are characterised by having specially constructed formal structures for camping. Those structures included either elevated camping platforms constructed on concrete beams or slightly elevated concrete/cement platforms constructed on the ground. Other facilities typically include a toilet in addition to a designated cooking area with table-like structures and seating made of cement and bricks [Fig. 2(b)].

#### Measurement of biophysical impacts

**Figure 3:**
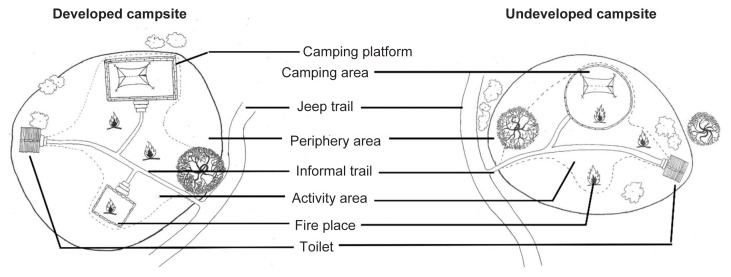
Generalised schematic diagram of developed and undeveloped campsites.

The assessment of biophysical impacts was carried out between the months of September and December 2016, during the dry season. Sketch maps of the selected campsites were initially drawn, indicating the campsite boundary, access route, location of main structures, and other important landmarks (Fig. 3). Based on the location of major infrastructure and the degree of ground exposure due to human activities, each campsite was considered to have two zones, an activity area and the periphery area. For the purposes of this study, the activity area was defined as the exposed area devoid of vegetation or litter cover due to human activities. The periphery area was defined as the outer area of the campsite where the ground has some vegetation cover or litter cover mostly not in the vicinity of camper activities, however signs of management activities by park management was evident. The total campsite area consisted of both the activity and periphery areas.

Site measurements followed the variable radial transect method (Marion 1995; Smith & Newsome 2002). The first transect was placed along the longest diameter axis of the campsite across the arbitrary centre point, and the number and placement of other transects were dependent on the shape of the campsite. Soil compaction was measured using a pocket penetrometer along transects at a 5 m interval from the arbitrary centre point of the campsite and in an adjacent, similar, undisturbed control site. In places where organic litter or an organic soil layer was present, it was carefully removed before compaction measurements and the compaction assessments were conducted in the mineral soil horizon. All the campsites were located close to a riverbank comprising alluvial soils, and compaction readings were considered to be independent of the soil type as the campsites had similar soils (Cooray *et al*. 1982). Hence, soil compaction data from campsites from each NP were pooled for statistical analysis.

A width of 2.5 m either side of transects was considered suitable for visual counts of litter (classified as biodegradable and non-biodegradable) and human damage to trees was also documented. Measurements of litter were expressed as “litter pieces or piles encountered per meter of transect”, and damage to trees was measured as “damage to trees encountered per meter of transect”. Root exposure was assessed along prominent informal trails leading to the water sources and key facilities in the site and expressed as “root exposure per meter of transect”. The campsites in the dry zone of Sri Lanka are subjected to flooding only during the short monsoon period. Hence root exposure on informal trails was assumed to be largely due to human use.

In assessing the degree of root exposure caused by anthropogenic activities, the degree of root exposure in peripheral areas of the campsite and control sites were compared. Additional information collected from the campsites included the area of the campsite, the extent of activity/exposed area and the periphery area, and the number of fireplaces/fire scars on the ground. The GPS coordinates of the campsite boundary, activity area, and periphery were recorded using a GARMIN^®^ etrex20 device, and the exposed ground area of campsites was calculated using the area measurement tool in Google Earth Pro^TM^. These parameters were further assessed using the impact rating scales described in Table 2 based on the work of Marion (1995) and Smith (2003).

To assess the loss of woody debris due to fuelwood collection around campsites, the forest fuel sampling method developed by Van Wagner (1968) and subsequently improved by Smith and Neal (1993) was applied. The method employed three survey lines each 10 m in length laid out to form an equilateral triangle placed near the boundary of the campsite. Two replicates were employed at each campsite, while for each campsite, an adjacent, similar, undisturbed control plot was also surveyed. The diameter of each piece of wood intercepting the survey line was recorded and categorised into one of the five size class being 0.5 cm–1 cm, 1.1 cm–2 cm, 2.1 cm–3 cm, 3.1 cm–4 cm and 4.1 cm and above.

As reported by Mackensen and Bauhus (1999, p. 2), there remains “no universally accepted standard definition” of the term “coarse woody debris”. Some studies have applied the term coarse woody debris to branches with a diameter greater than 10 cm (Woldendorp *et al*. 2002) or sticks and branches with a diameter greater than 7 cm (Woldendorp & Keenan 2005; Smith & Newsome 2002). However, other studies have classified sticks with diameters as small as 2.5 cm (Wei *et al*. 1997) and even 1 cm in diameter (Smith *et al*. 2012) as coarse woody debris. In contrast to Wei *et al*. (1997) and Smith *et al*. (2012), Woldendorp and Keenan (2005) and Smith and Newsome (2002) use the term “fine woody debris” for sticks with a diameter of less than 7 cm. That material could be broken by hand and used as kindling for camping fires (Smith & Newsome 2002). In a compromise between those varied positions, this study applies the term “coarse woody debris” to sticks and branches with a diameter greater than 3 cm and the term “fine woody debris” to sticks with diameters between 0.5 cm and 3 cm.

### The Visitor Survey to Assess Perceptions on Biophysical Impacts in Campsites

A structured questionnaire was used to gather information on visitor perceptions about biophysical impacts in campsites. The sample frame for the questionnaire survey included the visitors who have camped in the selected NPs of Sri Lanka during the period June 2015 to November 2016. Contact details of campers were collected from the booking centre at the Department of Wildlife Conservation. Visitors who have camped from June 2015 to November 2016 were contacted via telephone to administer the structured questionnaire. A total of 360 campers were contacted. Either the person who has done the booking or member of the camping party over 18 years old were interviewed. Individuals who complied with the request to participate in the survey were interviewed while those who declined to participate were treated as non-respondents. Respondents were given a range of attributes which asked to rate a particular issue on a 1 to 5 Likert scale (1 = very negative influence and 5 = very positive influence) to determine how biophysical impacts affect their overall camping experience. The survey further sought information on campers’ on-site behaviours and asked the respondents to rate their preference for selected campsite management actions on a Likert scale where 1 = strongly oppose and 5 = strongly support.

### Statistical Analysis of Biophysical Impacts

Relationships between the indicators of biophysical impacts described above and the usage of composites at the selected NPs were explored using Pearson’s correlation tests and independent sample *t*-tests (Berenson *et al*. 2006). One-way ANOVA tests were conducted to explore differences in the degree of soil compaction for different areas (i.e., activity areas, peripheral areas, and control areas) of campsites at each NP (Berenson *et al*. 2006). All statistical tests were assessed at the α = 0.05 level of significance.

## RESULTS

### Ground Exposure

The area of the studied campsites varied from 566 m^2^ to 1406 m^2^. The percentage exposed area (activity area) of campsites were between 22.13% and 52.28% of the total campsite area (Table 3). The positive relationship between usage level and ground exposure suggested by the Pearson’s correlation test was not statistically significant (*r =* 0.320, *p =* 0.368). Similarly, the independent sample *t*-test also found no significant difference between the percent (%) ground exposure at high and low use campsites (*t =* 0.87, *p =* 0.432).

### Root Exposure

Root exposure appeared more evident on major social trails traversed by campsite users. Severe root exposure was particularly evident on the high use campsites in Udawalawa NP (Table 3). However, the independent sample *t*-test found no significant difference between root exposure at high and low use campsites (*t =* −0.44, *p =* 0.683*)*. There was also no statistical significance for the Pearson’s correlation between days of occupancy of a campsite and severity of root exposure (*r =* −0.174, *p =* 0.630).

### Tree Damage

Bark peel-off, carvings on stems, removal of branch, trunk scars, and nail marks or nails embedded on tree trunks were the commonly observed human-induced damage to trees. The frequency and severity of damage as quantified in Table 2 appeared highly variable among campsites. Field observation suggested that such damage was more frequent in the activity areas of the campsite and in places where campers frequently congregate (e.g. cooking, sitting and tenting areas). According to the qualitative rating scale used (Table 2), tree damage observed at studied campsites varied from slight to moderate. However, the independent sample *t*-test showed no significant difference (*t* = 0.27, *p* = 0.793) between the frequency of tree damages in highly occupied and lesser occupied campsites (Table 3). Likewise, the relationship between tree damage and days of occupancy of campsites was not significant (*r =* 0.091, *p =* 0.803).

### Fire Scars on the Ground

Presence of fire scars outside the designated fireplaces was a common observation in 80% of the campsites investigated. These fire scars had been caused by campers making additional fireplaces for cooking purposes, and were often characterised by the presence of rocks, unburned wood debris, wood charcoal and ash. The number of fire scars on the ground varied between 2 and 6 and both high occupancy and less occupied campsites exhibited a high number of fire scars (Table 3).

### Littering and Cleanliness

Both biodegradable and non-biodegradable litter items were encountered as individual pieces or small piles. Biodegradable litter included items such as coconut shells, food waste and paper. Non-biodegradable litter consisted of plastic, glass, metal cans, polythene, clay pots, cigarette butts, styrofoam, ceramic and nylon ropes. The highly occupied campsites appeared less clean (Table 3) and the mean litter encounter rate at highly occupied campsites (1.47 ± 0.30) was higher than at lesser occupied campsites (1.17 ± 0.05). However, independent sample *t*-test (*t =* 2.29, *p =* 0.056*)* and the Pearson’s correlation test (*r =* 0.674, *p =* 0.047) provided *p*-values that are in the zone of uncertainty (i.e., *p* < 0.05). For that reason, no conclusion can be drawn as to the significance of the observed difference in the level of littering at the highly occupied sites.

### Soil Compaction and Penetration Resistance

The one-way ANOVA test revealed statistically significant differences among different user-areas of campsites in each NP (Table 4). Tukey’s post-hoc tests further confirmed the statistically significant differences among activity, peripheral and control areas (*p <* 0.05), indicating the dispersing nature of soil compaction impact away from the core area. As anticipated, activity areas of the campsites were more compacted than the periphery areas of the campsites. Mean soil compaction in the activity area of campsites ranged from 2.44 kg/cm^2^ to 3.75 kg/cm^2^, which was higher compared to periphery areas of campsites (Table 4). Peripheral areas of the campsites were significantly more compacted than control areas (*p <* 0.05*)*.

### Loss of Woody Debris

The most frequently recorded diameter size class of fuelwood from both control sites and campsites was the smaller pieces of fine woody debris between the diameters 0.5 to 1cm (Table 5). As evident in Table 6, independent sample *t*-tests revealed that the frequency of fine woody debris belonging to each diameter class at campsites is significantly less than that of control sites. While the *p*-values for the comparisons between the two classes of coarse woody debris (diameters greater than 3 cm) are inconclusive (*p* ≈ 0.05), the real-world context for that finding is that the low number and variability of the coarse woody debris found in the control areas has impacted the statistical result, rather than there being more coarse woody debris than fine woody debris (diameters 0.5 cm and 3 cm) found in and around the campsites. In general, these finding indicate a significant loss in the woody debris of all sizes classes near campsites.

### Visitor Perceptions on Biophysical Impacts on Campsites

#### Visitor experience regarding environmental impacts

Respondents were given a range of attributes and asked to rate them on a 1 to 5 Likert scale (where 1 = very negative influence, 3 = no influence, 5 = very positive influence) on how these attributes affected their overall camping experience during the most recent visit. Accordingly, ‘overall cleanliness’ was the most highly rated attribute by campers to affect their experience, followed by ‘presence of wildlife on or around the campsite’ and ‘availability of wood for firewood’ (Table 6). The current levels of solid waste disposal, vehicle-related impacts, vandalism and littering appeared to diminish camper experience.

#### Onsite visitor behaviours

Respondents in general responded positively to the first four statements in Table 7 with mean values of between 4.12 and 4.29 indicating that most campers agreed that their onsite behaviours were in accordance with the camping protocols and guidelines that apply at the selected NPs. Further, the respondents disagreed with the last six statements in Table 7, which cross-validated their responses for the first four statements. The neutral position of respondents (mean values 3.40 to 3.41) regarding their use of foot trails (other than those created by the park management) collection of firewood lying on the ground in the vicinity of their campsite, and only using the provided toilet facilities on campsites indicate that campers tend to deviate from required and desired camping behaviours. As highlighted below, thes failure to comply with camping protocols and desired onsite behaviours is probably due to the overall lack of suitable on-site facilities provided by management (Table 7).

## DISCUSSION

This study identified the main types of biophysical impacts of camping activities with respect to the degree of usage. The field procedures and methods used to assess the campsite impacts were adopted from established literature (Marion 1995; Smith & Newsome 2002; Smith *et al*. 2012; Eagleston & Marion 2017). Hence the findings of this study may be compared with impact situations of similar studies carried out elsewhere, allowing a more comprehensive interpretation and understanding of camping impacts in a regional and global context. Moreover, this study employed both field assessment of camping impacts coupled with a visitor survey. Such mixed methods have been employed in studies elsewhere (Smith & Newsome 2002), and the indicators identified in the visitor survey allowed a better understanding of the impacts of concern to visitors.

### Loss of Woody Debris

In this study, the loss of woody debris was assessed in selected campsites located in tropical dry-mixed evergreen forests of Sri Lanka, characterised by a prolonged dry period. There was a significant loss of woody debris (range of diameters considered) in campsites compared to control sites, indicating the extent of collection of firewood by campers. In forest recreation areas where campfires are permitted. Branches and logs of removable size are likely to be collected from a considerable area around campsites, resulting in the localised loss of woody debris from the forest floor and branch material from standing live and dead trees (Reid & Marion 2005; Smith *et al*. 2012). The woody debris on the forest floor are an important factor that determines the species richness of numerous specialised organisms including mosses, lichens, fungi and invertebrates, especially insects (Jabin *et al*. 2004; Jonsell *et al*. 2007; Hegetschweiler *et al*. 2009). Thus, long-term loss of woody debris in forest ecosystems can potentially cause significant ecological repercussions (Harmon *et al*. 1986; Driscoll *et al*. 2000; Newsome *et al*. 2013).

Loss of woody debris in and around campsites where campfires are allowed has been identified as a significant biophysical impact in temperate countries, which may require specific management interventions (Smith & Newsome 2002; Newsome *et al*. 2013). For instance, firewood is provided at formal campsites in certain protected areas in Australia as a management strategy to reduce the impact of firewood collection (Smith & Newsome 2002; Smith *et al*. 2012). In the context of camping in NPs in Sri Lanka, campers predominantly collect fuelwood from the vicinity of the campsite, although the removal of branches from dead or living trees is not allowed by mandate. According to the findings of the survey of campers, it was evident that most campers collected firewood lying on the ground from the vicinity of campsites, however, cutting and collecting firewood was also evident during the field assessments. Further, most visitors in our study did not concur with this management intervention of providing fuelwood by the park management, possibly indicating their desire to have a unique wilderness experience (Perera *et al*. 2012; Mallikage *et al*. 2018). Nonetheless, the effectiveness of such management interventions is contentious as reported by Smith and Newsome (2002) where they observed a significantly lower availability of coarse woody debris around campsites even when the firewood is provided by the park management. In contrast, Smith *et al*. (2012) reported that the provision of firewood for campers significantly reduced the extent of impact in relation to woody debris removal and tree damage in some of the protected areas in Western Australia.

### Impacts on Soils and Ground Vegetation Cover

In this study, the impacts to soils were predominantly assessed using soil compaction measured by penetration resistance. The spatial pattern of soil compaction was comparable with findings in the literature where the highest soil compaction being recorded from activity area where most human activities generally take place (Smith & Newsome 2002; Cole 2004; Reid & Marion 2005; Andrés-Abellán *et al*. 2005; Marion *et al*. 2016). The levels of soil compaction dispersed away from the core area, indicating the campsite impact area expansion due to human activities such as fuelwood collection (Smith *et al*. 2012).

Soil compaction measurements provide insight into the level of porosity of the soil, infiltration capacity, soil degradation, and soil erosion. However, soil compaction at studied campsites was at a level not hindering vegetation growth, even though there is a significant difference among soil penetrometer readings at activity, peripheral and control areas. In our study, the soil penetrometer readings obtained ranged between 0.31 kg/cm^2^ and 3.75 kg/cm^2^ (0.03 MPa and 0.37 MPa) which implied that the degree of soil consolidation was not dense with plant root growth not likely to be severely affected (Alessa & Earnhart 2000; Hazelton & Murphy 2007). Compaction of a campsite becomes an issue when water is no longer infiltrating into the soil and pools on the surface creating the necessity for visitors to excavate trenches around tents for drainage, thus increasing the risk of campsite erosion (Newsome *et al*. 2013). However, compacted soils especially on flat recreation sites and informal trails can be perceived as beneficial where there is no soil displacement and soil loss (Marion *et al*. 2016). Irrespective of the degree of compacted soils, vegetation growth in activity areas and along informal trails in campsites is compromised by trampling. However, soil compaction can be viewed as a positive aspect in campsite impact management strategies that focus on concentrating human activities to a limited high use of the campsite.

Erosion loss in campsites was assessed by measuring root exposure and the total exposed ground area in the campsite. Root exposure in studied campsites varied from slight to severe. However, no correlation was found between the degree of root exposure and the level of campsite usage. Likewise, there was no difference in the percentage of exposed bare ground observed at high and low use campsites.

Results of the visitor survey further highlight that signs of vegetation loss, trampling, erosion of trails and riverbanks due to human activity, and vehicle-related impacts on soils negatively affect visitor experience. This highlights the importance of monitoring and subsequent management of soil and vegetation-associated impacts at campsites, as neglecting these aspects over time, could lead to negative visitor experiences (Perera & Vlosky 2013). Accordingly, visitor approved potential management interventions such as the education of campers on minimal impact use and camping techniques, improved onsite interpretation and awareness, and providing minimal structures to stabilise riverbanks are vital management actions. Mallikage *et al*. (2018) reported that ‘to be in a natural setting’ is a major motivation of camping in NPs of Sri Lanka. This may explain why visitors are not perceiving poorly maintained walk trails in campsites as a negative attribute.

#### Impacts of campfire scar proliferation

The literature on campfire impacts reveals an extensive list of resource damage attributed to campfires including fire site proliferation, overbuilt fire sites, user constructed seating arrangements, fuelwood depletion, vegetation damage, charred rocks and tree roots, ash as well as the presence of charcoal build-up, semi-melted plastic, glass, and metal trash (e. g. Reid & Marion 2005; Hegetschweiler *et al*. 2009; Smith *et al*. 2012; Jackson 2016). Observations made in the present study indicate a proliferation of fireplaces/fire scars in the studied campsites. Such an occurrence could be attributed to a lack of monitoring and the absence of well-directed guidelines. The field observations contrast with the results of the visitor survey, which suggest that majority of campers used only the designated areas for camping**-**related activities. However, observed evidence of burning of polythene and plastic waste at fireplaces detected in this study suggest attention needs to be given to litter management at campsites in Sri Lanka.

### Littering and Waste Disposal

Non-biodegradable waste such as glasses, plastics, and polythene can pose a potential risk to wild animals (Newsome *et al*. 2013). It was further observed that, due to food waste disposal, wild animals such as toque macaques (*Macaca sinica*) and wild boars (*Sus scrofa*) are frequently attracted to campsites, causing nuisance and potential risks to visitors.

Visitor survey outcomes suggest that littering/solid waste disposal is a concern affecting visitor satisfaction at studied campsites. Regardless of the numerous ecological consequences, litter accumulation at frequently used campsites is a negative experience for visitors (Hegetschweiler *et al*. 2009). In this study, visitors ranked ‘overall cleanliness’ of the campsite as the most important factor affecting visitor satisfaction. Hence, preserving recreational areas as attractive and clean should be an aim of NP management for sustained quality experience in natural settings. Even though the current user policy encourages visitors to take their litter with them without disposing or burning onsite, the positive uptake of these guidelines remains questionable as indicated by the study results.

### Tree Damage

Tree damage was observed at all of the studied campsites at varying levels. Based on the rating scale used in this study (Table 3), it could be interpreted that the tree damage varied from slight to moderate. In addition, it was apparent that tree damage is more concentrated in highly used areas within camping areas such as tenting and cooking places, riverbanks and in places with seating arrangements, with no difference in the extent of damage recorded at high and low use campsites. However, the results suggest that campers were less sensitive to tree damage at studied campsites compared to other evidence of vandalism (Table 7). This may be since evidence of vandalism is more noticeable even though its occurrence maybe minimal or restricted to just a single case. Visitor behaviour attributes used in the questionnaire further revealed that not all campers cause tree damage and vandalism. In general, a few irresponsible campers account for much of the damage (Marion & Reid 2007; Littlefair & Buckley 2008; Pickering 2010).

#### The impact of site hardening

Recent studies suggest that site hardening measures such as the installation of camping platforms at major campsites can manage campsite impacts (Dixon & Hawes 2015; Dixon 2017). The sample size (four developed campsites and six undeveloped campsites) and the confounding effect of usage levels did not warrant a meaningful comparison of biophysical impacts between developed and undeveloped campsites. If a site is more frequently subject to recurring disturbances, biophysical impacts may be more prominent as less time is available for the ecosystem to recover naturally (Cole & Monz 2004; Growcock 2005; Arredondo *et al*. 2021). Although it can be argued that the elevated camping structures in developed campsites would reduce the area of impact, a cursory examination of the biophysical impacts reported in this study, such as the percentage exposed area and root exposure along informal trails did not provide any conclusive evidence to support this contention. The literature further suggests that provision of inviting facilities, including camping platforms can result in a concentration of visitor camping use on a smaller number of campsites (Dixon 2017). In contrast, the developed campsites examined in this study were mostly in the “low use” category, suggesting the visitor preference for campsites in the Sri Lankan context may be overshadowed by the visitor type and preference and key biodiversity features of the park rather than the campsite facilities (Senevirathna & Perera 2013; Mallikage *et al*. 2018).

### The Impetus for Integrated Monitoring and Management of Campsites

The visitor survey examined visitor experience regarding environmental impacts that have been identified in the study and how a range of impacts affect their quality of experience. Majority of respondents considered overall cleanliness, the presence of wildlife, availability of wood for firewood and sanitary facilities as the most important factors for their satisfaction. Solid waste disposal, vehicle-related impacts, vandalism, and littering were the attributes that negatively influence camper experience. These aspects need attention along the lines as suggested by Marion *et al*. (2020) in order to ensure that management can deliver sound environmental protection and a higher quality visitor experience. Although biophysical impacts at developed and undeveloped campsites were considered during this study, the nature of development or the infrastructure at campsites did not seem to have any effect on the biophysical parameters that were evaluated via actual measurements and the visitor questionnaire.

Based on the survey results, overall cleanliness at campsites plays a distinct role in creating a positive visitor experience and it is an indicator which should be prioritised by park management when monitoring campsites. Visitors also favoured observing wild animals around campsites. However, it was evident that some animals have become habituated to humans at campsites and the improper waste disposal practices (food waste) and visitor behaviours such as feeding wildlife have attracted these animals to the vicinity of campsites. Even though current park management strategies prohibit disposing waste at campsites it was revealed that some campers dispose food waste onsite and burn polythene and plastic waste before leaving the NP. There is some evidence that campers with comparatively lesser education levels tend to engage in such environmentally inappropriate activities (Mallikage *et al*. 2018). Visitor policies, therefore, should be amended to promote responsible visitor behaviours while enforcement of guidelines and interpretation aimed at educating visitors can also play a key role. Unnecessary use of fires and encouraging visitors to bring firewood from outside can be encouraged to prevent the adverse impacts of loss of woody debris around campsites. However, as noted previously such measures were not favoured by campers.

### Limitations of the Study

The selected biophysical impacts were assessed only during one climatic season, which was the dry season when campsite visitation and usage are highest. Hence, certain biophysical impacts such as ground exposure, social trails, littering, and garbage disposal may be more observable during the dry period due to both climatic and visitor induced factors. Similarly, certain biophysical impacts such as trail erosion and root exposures can be intensified during the rainy season. Moreover, low levels of visitor activity during the wet season, coupled with favourable conditions for vegetation growth, may provide a recovery period for campsites. Therefore, ongoing year-round monitoring may provide a more accurate picture of the severity and dynamics of biophysical impacts on campsites in the selected NPs. Frequent movement of animals across some of the studied campsites made it difficult to attribute all biophysical impacts solely to human activities. As such, there is a need for more research to distinguish the actual biophysical impacts of camping on protected areas in Sri Lanka.

The visitor survey was conducted using phone interviews to sample campers who engaged in camping at selected campsites within June 2015 to August 2016. Hence, the survey may suffer from inherent recall bias involved in data gathering. Nonetheless, only those who have camped recently (within 12 months of the start of survey) were selected in order to minimise the effect of recall bias.

## CONCLUSION

In this study, biophysical parameters were assessed pertaining to camping in selected national parks in the dry zone of Sri Lanka. Among the biophysical parameters evaluated, a significant loss in woody debris was observed at all campsites compared to undisturbed areas. Soil compaction was higher in areas of campsites where visitor activities are concentrated. However, with increased visitations in the future, there’s a possibility that soil compaction would reach levels that hinder plant growth if necessary management interventions are not carried out. Possible management actions include strict enforcement of visitor policies, education and awareness for campers on minimal impact camping, and provision of signs/directions on campsites to indicate designated use areas. The proliferation of fireplaces, fire scars on the ground and littering/waste disposal appear to be emerging biophysical impacts of concern in the studied campsites and require management attention. Both littering and tree damage parameters suggest that these impacts are directly related to visitor behaviour rather than the visitation level. Thus, regulating visitor behaviour through awareness and implementation of visitor policies should be seriously considered by the park management.

This study employed a mixed method approach comprising both physical and visual assessment of campsite impacts coupled with a visitor survey to identify impacts of concern to visitors. Such an integrated approach is useful in the assessment and monitoring of biophysical impacts on campsites. The campsite biophysical impact profile documented through field observations and visitor survey established useful baseline information for the long-term monitoring and management of campsites in the dry-zone NPs of Sri Lanka.

## Figures and Tables

**Table 1:** Categorisation of examined campsites (Average monthly occupancy in days).

Name of the campsite	Site code	Features	Type	Average monthly occupancy	Usage level
Udawalawe (UW)					
Alimankada	UW1	Camping ground, toilet	Undeveloped	7.53	High
Pilimaddhara	UW2	Camping ground, toilet	Undeveloped	7.18	High
Pranshadhara	UW3	Camping ground, toilet	Undeveloped	9.21	High
Wasgamuwa (WG)					
Madapitiya 1	WG1	Elevated platform, cooking/dining, toilet	Developed	4.59	Low
Madapitiya 2	WG2	Elevated platform, cooking/dining, toilet	Developed	4.66	Low
Mahaweli 1	WG3	Elevated platform, cooking/dining, toilet	Developed	7.63	High
Mahaweli 2	WG4	Elevated platform, cooking/dining, toilet	Developed	5.99	Low
Wawulabe	WG5	Camping ground, toilet	Undeveloped	3.50	Low
Yala (YL)					
Kosgasmankada	YL1	Camping ground, toilet	Undeveloped	15.23	High
Nugasewana	YL2	Camping ground, toilet	Undeveloped	10.82	High

**Table 2:** Impact rating scales for root exposure, tree damage, and ground exposure.

Parameter	Rating	Descriptor	Explanation
Root exposure	1	None	Other than naturally exposed root formations (such as buttresses)
	2	Slight	Only the tops of major roots are slightly exposed
	3	Moderate	Tops of a majority of the major roots exposed or severe exposure of tops and sides of one or two major roots
	4	Severe	Tops, sides, and undersides of many of the major roots exposed

Tree damage	1	None	Other than natural causes
	2	Slight	Trees with only superficial scars and small branch cut-offs or broken
	3	Moderate	Trees with superficial scars, small branch cut-offs or broken, one or two trees with deep scars, nails, nail holes
	4	High	Large branches cut off or broken, nail holes, nails, deep scars/extensive mutilation

Ground exposure	1	Low	Less than 25% ground exposed
	2	Moderate	25% to 50% ground exposed
	3	High	More than 50% of the ground exposed

Cleanliness	1	Clean	No signs of inappropriate waste disposal, well-maintained designated fireplace
	2	Acceptable	Some signs of inappropriate waste disposal; less than 25 pieces/small piles of litter mainly in periphery area, use of the designated fireplace, but charcoal and burned wood pieces scattered around
	3	Poor	Signs of inappropriate waste disposal; 25 to 50 pieces/small piles of litter both in activity and periphery areas, including 1 or 2 signs of human waste disposal; up to 2 fireplaces other than the designated ones with charcoal and burned wood pieces scattered around
	4	Terrible	Obvious signs of inappropriate waste disposal; over 50 pieces/small piles of litter both in activity and periphery areas, including 3 or more signs of human waste disposal; 3 or more fireplaces other than the designated ones with charcoal and burned wood pieces scattered around

**Table 3:** Percentage exposed area of campsites.

Campsite	Usage level	Total area (m^2^)	Exposed area		Root exposure		Tree damages		No. of fireplaces	litter/m	Cleanliness
		
			(%)	Rating	Frequency/m	Rating	Frequency/m	rating			
UW1	High	1161	43.67	2	0.13	2	0.15	2.11	4	1.55	3
UW2	High	1369	53.11	3	0.20	3	0.37	2.23	6	1.47	3
UW3	High	1387	22.13	1	0.07	4	0.31	2.32	4	1.06	2
WG1	Low	1406	14.65	1	0.40	2	0.43	2.49	5	1.14	2
WG2	Low	1223	26.43	2	0.15	2	0.08	3.00	5	1.24	2
WG3	High	894	36.02	2	0.24	3	0.27	2.17	2	1.96	3
WG4	Low	750	38.27	2	0.09	2	0.24	2.53	6	1.14	3
WG5	Low	725	52.28	3	0.17	2	0.29	2.75	3	1.16	2
YL1	High	609	41.71	2	0.25	2	0.58	2.27	5	1.49	3
YL2	High	566	53.18	3	0.13	2	0.05	2.50	5	1.28	3

**Table 4:** Significance of soil compaction at campsites according to the area considered;

NP	Area	Sample size	Mean compaction (kg/cm^2^)	*F*-value	*p*-value
UW	Activity	12	3.27 ± 0.91	32.53	<0.001*
	Periphery	12	1.81 ± 0.96		
	Control	12	0.67 ± 0.37		
WG	Activity	12	2.92 ± 0.69	53.55	<0.001*
	Periphery	12	1.63 ± 0.71		
	Control	12	0.44 ± 0.22		
YA	Activity	8	3.31 ± 0.93	55.56	<0.001*
	Periphery	8	0.84 ± 0.27		
	Control	8	0.59 ± 0.19		

*Note*:

* Statistical significance at α = 0.05 level

**Table 5:** Comparison of mean frequencies of woody debris around campsites with control plots (Diameter class in cm).

Diameter class	Site	Sample size	Mean	*t*-value	*p-*value
0.5 cm–1 cm	Campsite	20	18.20 ± 5.29	−3.04	0.012*
	Control	10	31.60 ± 13.41		
1.1 cm–2 cm	Campsite	20	2.20 ± 2.31	−4.12	0.002*
	Control	10	11.10 ± 6.64		
2.1 cm–3 cm	Campsite	20	0.35 ± 0.75	−3.96	0.003*
	Control	10	3.50 ± 2.46		
3.1 cm–4 cm	Campsite	20	0.05 ± 0.22	−2.27	0.049*
	Control	10	1.50 ± 2.01		
Above 4 cm	Campsite	20	0.05 ± 0.22	−2.27	0.049*
	Control	10	± 1.03		

*Note*:

* Statistical significance at α = 0.05 level

**Table 6:** Means and standard deviations for attributes affecting the overall camping experience (rated on a scale where 1 = very negative influence and 5 = very positive influence).

Attribute	*N*	Mean	SD
Overall cleanliness	202	3.61	0.96
Presence of wildlife on or around a campsite	200	3.58	0.77
Availability of wood for firewood around a campsite	202	3.38	0.87
Poorly maintained walk trails	113	3.02	0.64
Signs of vegetation loss	169	2.99	0.85
Erosion of trails due to human activity	142	2.91	0.74
Erosion of riverbanks due to human activity	185	2.86	0.73
Sanitary facilities	202	2.86	0.99
Presence of invasive plant species	110	2.82	0.96
Trampling of vegetation	162	2.78	0.80
Tree damage	153	2.73	0.79
Presence of litter	201	2.69	1.01
Vandalism activities	148	2.68	0.60
Vehicle-related impacts	200	2.54	0.89
Solid waste disposal	196	2.50	0.86

**Table 7:** Means scores for onsite visitor behavioural attributes (rated on a scale where 1 = strongly disagree and 5 = strongly agree) (*N* = 202).

No	Attribute	Mean	SD
	During the camping period, I…		
1	observed nature and wildlife thoroughly.	4.29	0.67
2	collected and brought all polythene and plastic waste.	4.24	0.49
3	followed the instructions/ guidelines provided during the tour by the guide.	4.23	0.51
4	used only the designated areas for camping-related activities.	4.12	0.54
5	used foot trails other than the trails created by the park management.	3.41	0.74
6	collected firewood (lying on the ground) from the vicinity of the campsite.	3.41	0.94
7	used only the provided campsite toilet facilities.	3.40	1.06
8	disposed food waste to nearby forest or water body so that animals/fish can feed on them.	2.82	1.21
9	disposed food waste in designated areas at the campsite.	2.77	1.09
10	buried food/organic waste.	2.61	1.14
11	used nearby forest to cut and collect firewood.	2.42	1.08
12	fed wildlife.	2.12	0.91
13	burnt all polythene and plastic waste items before leaving the site.	2.04	0.89
14	buried non-biodegradable waste (e.g., plastic, polythene).	1.81	0.60
